# Structural and dynamic properties of the YTH domain in complex with N^6^‐methyladenosine RNA studied by accelerated molecular dynamics simulations

**DOI:** 10.15302/J-QB-022-0297

**Published:** 2023-03-01

**Authors:** Mingwei Li, Guanglin Chen, Zhiyong Zhang

**Affiliations:** ^1^ MOE Key Laboratory for Membraneless Organelles & Cellular Dynamics National Science Center for Physical Sciences at Microscale Division of Life Sciences and Medicine, and Biomedical Sciences and Health Laboratory of Anhui Province University of Science and Technology of China Hefei 230026 China; ^2^ Department of Physics University of Science and Technology of China Hefei 230026 China

**Keywords:** RNA methylation, YTH‐m^6^A3 RNA, principal component analysis (PCA), binding free energy, contact‐based PCA

## Abstract

**Background:**

N^6^‐methyl adenosine (m^6^A) modifications of mRNA and long non‐coding RNA (lncRNAs) are known to play a significant role in regulation of gene expression and organismal development. Besides writer and eraser proteins of this dynamic modification, the YT521‐B homology (YTH) domain can recognize the modification involved in numerous cellular processes. The function of proteins containing YTH domain and its binding mode with N^6^‐Methyladenosine RNA has attracted considerable attention. However, the structural and dynamic characteristics of the YTH domain in complex with m^6^A RNA is still unknown.

**Method:**

This work presents results of accelerated molecular dynamics (aMD) simulations at the timescale of microseconds. Principal component analysis (PCA), molecular mechanics generalized Born surface area (MM/GBSA) calculations, contact analysis and contact‐based principal component analysis (conPCA) provide new insights into structure and dynamics of the YTH‐RNA complex.

**Results:**

The aMD simulations indicate that the recognition loop has a larger movement away from the binding pocket in the YTH‐A3 RNA than that in the YTH‐m^6^A3 RNA. In aMD trajectories of the apo YTH, there is a significant close‐open transition of the recognition loop, that is to say, the apo YTH can take both the closed and open structure. We have found that the YTH domain binds more favorably to the methylated RNA than the non‐methylated RNA. The per‐residue free energy decomposition and conPCA suggest that hydrophobic residues including W380, L383‐V385, W431‐P434, M437, and M441‐L442, may play important roles in favorable binding of the m^6^A RNA to the YTH domain, which is also supported by aMD simulations of a double mutated system (L383A/M437A).

**Conclusion:**

The results are in good agreement with higher structural stability of the YTH‐m^6^A RNA than that of the YTH‐A3 RNA. The addition of a methylation group on A3 can enhance its binding to the hydrophobic pocket in the YTH domain. Our simulations support a ‘conformational selection’ mechanism between the YTH‐RNA binding. This work may aid in our understanding of the structural and dynamic characteristics of the YTH protein in complex with the methylated RNA.

## Abstract 2

**Author summary:** N^6^‐methyl adenosine (m^6^A) modifications play an important role in the regulation of RNA decay and gene expression at the post‐transcriptional level. The YT521‐B homology (YTH) domain‐containing proteins can recognize m^6^A in numerous cellular processes. However, the binding mode of the YTH domain to m^6^A RNA is still unknown. In this work, we present an atomic description of the YTH‐m6A RNA complex using computer simulations. The results have revealed the molecular mechanism of the RNA binding to the YTH domain. Our findings may be helpful for understanding the structural and dynamic characteristics of the YTH‐m6A RNA complex.

## INTRODUCTION

RNA methylation of internal adenosine to form N^6^‐methyladenosine (m^6^A) is the most abundant epigenetic modification of all higher eukaryotic genomes [1], including mRNA and long non‐coding RNAs (lncRNAs) [2]. In mRNA, m^6^A is found to locate in the 3′‐UTR and stop codons, which suggests an important role in the regulation of RNA decay and gene expression at the post‐transcriptional level [3‒5], including affecting the translation status [6] and modulating disease aetiology [7]. While methyltransferase complex, such as METTL3–METTL14, could serve as the “writer” [8,9] and demethylases like FTO and ALKBH5 act as the “eraser” [10,11] of the m^6^A modification, the YT521‐B homology (YTH) domain‐containing proteins are named as the “reader” that could recognize and selectively bind N^6^‐methyadenosines RNA, and then control RNA life in a methylation‐dependent manner [12‒14].

The YTH domain [14] family known to recognize and bind single‐stranded RNA consists of five proteins (YTHDF1‐3 [4], YTHDC1 [4] and YTHDC2 [15]) in human cells. The first protein containing a YTH domain is the *Rattus norvegicus* protein YT521‐B (alternative name YTHDC1) [16,17]. The structure of the YTH domain of rat YT521‐B (residues 347‒502) in complex with N6‐methylated RNA (5′‐UGm^6^ACAC‐3′) has been solved by solution nuclear magnetic resonance (NMR) spectroscopy [18]. Previous studies have shown that the YTH domain has an obviously higher binding affinity with the m^6^A RNA than the non‐methylated RNA [18‒21]. The YTH domain could form a buried hydrophobic binding pocket that accommodates the m^6^A, involving hydrogen bonds and hydrophobic contacts [18‒20,22,23]. These findings have explained preferential recognition of UGm^6^ACAC sequences and showed the binding mode between the YTH domain and the methylated RNA. However, detailed structural and dynamic studies are required to better understand the mechanism of its recognition and binding characteristics.

To date, little structural and dynamic information on YTH domain in complex with the m^6^A RNA is known since it is not visible from a single static structure. Molecular dynamic (MD) simulations have been used to examine the molecular recognition mechanism between the reader domain of YTHDC1 from *Homo sapiens* and m^6^A‐containing RNA, and the results show that the m^6^A contributes to the stable binding through its interactions with residues involving an aromatic cage [24]. YTHDF1‐3 proteins have also been investigated by atomistic simulations. The recognition loop containing aromatic residues of m^6^A binding pocket has pronounced flexibility, which can take different conformations and facilitate the binding [25].

In this study, we focus on the YTH domain of YT521‐B in complex with the m^6^A RNA (5′‐UGm^6^ACAC‐3′). By utilizing accelerated MD (aMD) simulations and post‐processing analysis, the structural and dynamic characteristics and molecular mechanism of the YTH domain in complex with m^6^A RNA are revealed.

## RESULTS

### Structural properties of m^6^A recognition by the YTH domain

**Figure 1:**
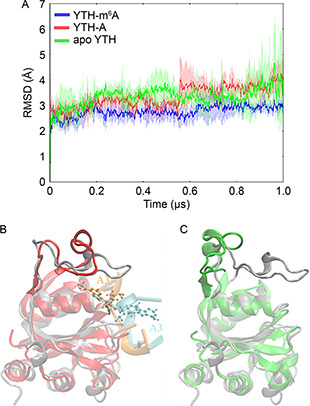
**Conformational changes of the YTH domain in complex with the m^6^A3/A3 RNA, and the apo form.** (A) Time evolution of RMSD in the YTHm6A3 RNA (blue), the YTH‐A3 RNA (red), and the apo YTH (green). The RMSD values were computed using Ca atoms of the protein, with respect to the starting structure. For each system, the average RMSD values and their standard deviations were calculated over the three independent aMD simulations. (B) Comparison between the initial structure of YTH‐A3 RNA and a representative structure in the aMD simulations. For the initial structure, the protein is colored by sliver and the RNA is colored by orange. For the representative structure, the protein is colored by red and the RNA is colored by cyan. The A3 is displayed in ball‐and‐stick. (C) Comparison between the initial structure of the apo YTH and a representative structure in the aMD simulations. The initial structure is colored the same as that in (B). For the representative structure, the protein is colored by green.

To obtain statistically meaningful results, for each system (the apo YTH, the YTH‐m^6^A3 RNA and the YTH‐A3 RNA), three independent 1‐μs aMD simulations were performed. From each simulation, a trajectory containing 1000 conformations extracted every 1 ns were used for analysis. Root mean square deviations (RMSD) of conformations in aMD trajectories to the starting structure can indicate overall conformational changes of the system ( Fig.1). The average RMSD and standard deviations of each system were calculated over the three independent trajectories, using C_α_ atoms of the protein. RMSD values of the YTH‐m^6^A3 RNA are essentially stabilized at around 2.8 Å ( Fig.1, blue). However, during the aMD simulation of the YTH‐A3 RNA, the average RMSD values are generally larger than those in the YTH‐m^6^A3 RNA ( Fig.1, red). In one of the three aMD simulations, the recognition loop is open that makes the binding pocket exposed, and thus the A3 is release (a representative structure is shown in Fig.1). In the other two aMD simulations of the YTH‐A3 RNA, the complex is relatively stable. The average RMSD values and their fluctuation of the apo YTH ( Fig.1, green) can be even larger than those of the YTH‐A3 RNA. In the two aMD simulations of the apo YTH, the recognition loop is widely open (a representative structure is shown in Fig.1), whereas the loop keeps closed in one aMD simulation. The results suggest that the close/open of the recognition loop may determine the binding/release of the m^6^A3 or A3. In the initial protein structure ( Fig.1 and C, gray), the recognition loop covers the binding pocket, and the ligand is deeply inserted in the pocket. When the loop uncovers, the ligand would release.

### Conformational sampling of the YTH domain

To identify collective motions of the recognition loop, we performed principal component analysis (PCA) [26] on the apo YTH. The three aMD trajectories were combined, and thus PCA was conducted on 3000 conformations using all the C_α_ atoms. Generally, a few PCA modes with the largest amplitude (called the essential PCA modes) describe collective motions in the protein that may be functionally relevant [27]. The first PCA mode (PC1) of the apo YTH contributes about 26.2% to the total fluctuation, whereas the second mode (PC2) has a contribution of about 15.3%.

**Figure 2:**
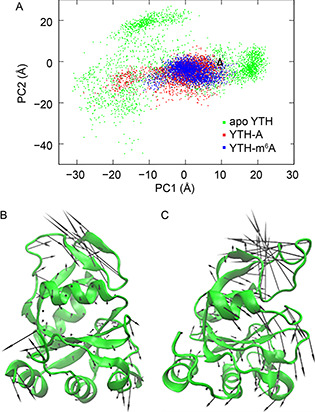
**Conformational sampling of the YTH systems.** (A) Projection of the different aMD trajectories onto the first two PCA modes (PC1 and PC2) defied by the apo YTH. Blue: the YTH‐m^6^A3 RNA, red: the YTH‐A3 RNA, and green: the apo YTH. The up‐triangle indicates the location of the initial structure. (B) Collective motion along the PC1 of the apo YTH. The conformations with the most negative and the most positive projection values on PC1 were superimposed, and then arrows were drawn between the corresponding C_α_ of the two conformations. One conformation was hidden. (C) Collective motion along the PC2 of the apo YTH.

Projecting trajectories onto the subspace spanned by the essential PCA modes is a good way to visualize sampled conformational space and reveal major motions in a simulation. The aMD trajectories of the apo YTH were projected onto a 2D subspace defined by the PC1 and the PC2 ( Fig.2, green). For each mode, we took conformations with the most negative and the most positive projection values, superimposed them, and drew arrows between the corresponding atoms. Along the PC1, the recognition loop shows a significant close‐open motion ( Fig.2), whereas along the PC2, the loop also has a twist motion ( Fig.2). It can be seen that the apo YTH samples a large conformational space ( Fig.2, green). The recognition loop may take different conformations, which can be widely open or even closer than the initial structure ( Fig.2, up‐triangle).

We then project of the aMD trajectories of the YTH‐m^6^A3 RNA and the YTH‐A3 RNA onto the same essential subspace. The YTH‐m^6^A3 RNA only samples a limited region ( Fig.2, blue) and the conformations are relatively stable with the closed recognition loop. For the YTH‐A3 RNA, although many conformations cover the same region as the YTH‐m^6^A3 RNA, there are some conformations sampled along the PC1, with the opening recognition loop ( Fig.2, red). It should be noted that, both the YTH‐m^6^A3 RNA and the YTH‐A3 RNA do not sample the twist motion of the recognition loop along the PC2 of the apo YTH. The conformational sampling on the essential subspace indicates that, the un‐methylated adenosine could weaken the binding between the YTH domain and RNA by promoting the opening of the recognition loop. Furthermore, our results support a “conformational selection” mechanism upon the binding of RNA to the YTH domain.

### Molecular mechanism of the RNA binding to the YTH domain

The MM/GBSA (molecular mechanics generalized Born surface area) method [28] was used to estimate binding free energy between the protein and the RNA using the aMD trajectories. The average binding free energy between the m^6^A3 RNA and the YTH is ‒137.5±21.3 kcal mol^−1^, and the value between the A3 RNA and the YTH is ‒111.0±18.2 kcal mol^−1^. That is to say, the m^6^A3 RNA can bind with the YTH more favorably than the A3 RNA.

**Figure 3:**
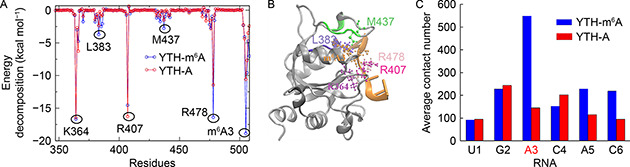
**Interactions between the YTH domain and the m^6^A3/A3 RNA.** A) Per‐residue free energy decomposition of the YTH‐RNA complexes. Blue: the YTH‐m^6^A3 RNA, and red: the YTH‐A3 RNA. (B) The m^6^A3 and some residues contributing to the binding are displayed in the initial structure of the YTH‐m^6^A3 RNA. The m^6^A3, K3^6^4, L383, R407, M437, R478 are shown in ball‐and‐stick and colored by orange, purple, violet, magenta, green, and pink, respectively. The other hydrophobic residues within 380.385 are colored by violet as L383, and those within 431.442 are colored by green as M437. (C) Average contact numbers between the YTH domain and the m^6^A3 RNA (blue), and the A3 RNA (red), during the aMD simulations.

We have also computed per‐residue decomposition of the binding free energy. In the YTH domain, there are some groups of residues contributing to the binding free energy, which are residues 362‒366, 380‒386, 407, 431‒442, and 469‒479 ( Fig.3). These residues with positive charge, such as K364, R407 and R478, were reported to interact with the phosphate oxygens in the RNA [18]. They do not belong to the recognition loop and has no contacts with the methylation group in the m^6^A3 ( Fig.3). The other two groups of residues (380‒386 and 431‒442) contain quite some hydrophobic residues including W380, L383, P384, V385, W431, V432, L433, P434, M437, M441 and L442. They form a hydrophobic pocket that binds favorably to the m^6^A3. The residues 431‒442 are located in the recognition loop that cover the pocket, and the residues 380‒386 are at the bottom of the pocket. In the RNA molecule, the m^6^A3 does have the largest contribution to the binding free energy ( Fig.3).

Furthermore, the average contact number between each nucleotide and the YTH domain was computed for the YTH‐m^6^A3 RNA and the YTH‐A3 RNA ( Fig.3). A contact is defined when the distance between a heavy atom in a protein residue and another heavy atom in a nucleotide is within 6.0 Å. In the YTH‐m^6^A3 RNA, the nucleotides m^6^A3, A5, and C6 have more contacts with the YTH domain ( Fig.3, blue) than those in the YTH‐A3 RNA ( Fig.3, red). Among them, m^6^A3 forms the largest number of contacts with the protein. The results suggest that m^6^A3 makes the biggest contribution to the binding between the YTH domain and m^6^A3 RNA. Interestingly, the two neighbors of m^6^A3, G2 and C4, show decreased number of contacts compared to their correspondences in the YTH‐A3 RNA.

**Figure 4:**
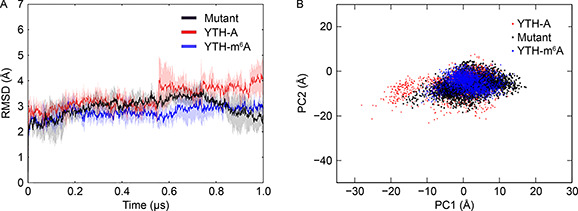
**Conformational changes of the YTH domain induced by mutations.** (A) Time evolution of RMSD in the mutYTHm^6^A3 RNA (black). The RMSD values of the YTH‐m^6^A3 RNA (blue) and the YTH‐A3 RNA (red) are also shown for comparison. (B) Projection of the mutYTH‐m^6^A3 RNA trajectories (black) onto the essential subspace defined by the apo YTH. The projection of the YTH‐m^6^A3 RNA (blue) and the YTH‐A3 RNA (red) trajectories are also shown for comparison.

The aforementioned results indicate that those hydrophobic residues play an important role in favorable binding to the m^6^A3 RNA. As a test, we have mutated two residues (L383A and M437A) in the YTH‐m^6^A3 RNA, and conducted three independent 1‐μs aMD simulations. The average binding free energy of the mutYTH‐m^6^A3 RNA is about ‒110. 2±20.5 kcal mol^‒1^. Comparing to the binding free energy of ‒137.5±21.3 kcal mol^‒1^ in the YTH‐m^6^A3 RNA, the interactions between the mutated YTH and the m^6^A3 RNA may become weaker. The average RMSD values and their standard deviations of the mutant ( Fig.4, black) are generally larger than those in the YTH‐m^6^A3 RNA ( Fig.4, blue), but smaller than those in the YTH‐A3 RNA ( Fig.4, red). By projecting trajectories of the mutant onto the essential subspace, we can see a small number of conformations moving to the left along the PC1 that means they become open ( Fig.4, black). The aMD simulations of the mutant support that, the contacts between the hydrophobic residues and m^6^A3 play an important role in stability of the YTH‐m^6^A3 RNA.

### Contact‐based PCA (conPCA) of the YTH‐RNA complexes

**Figure 5:**
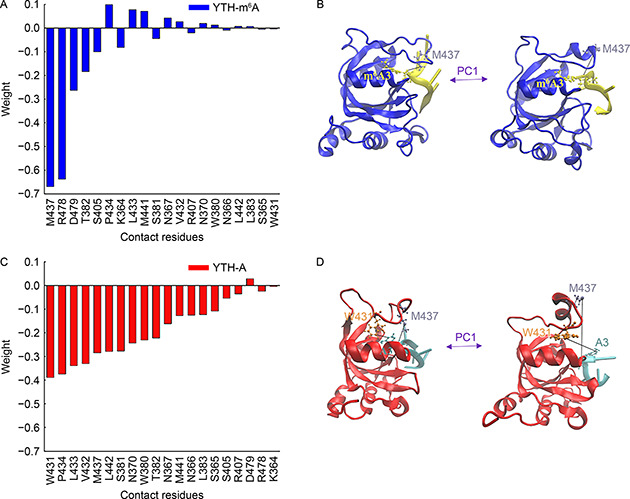
**Dynamically important contacts identified by conPCA.** (A) Eigenvector components of the PC1 obtained from conPCA of the YTH‐m^6^A3 RNA. (B) Representative structures of the YTH‐m^6^A3 RNA discriminated by the PC1. (C) Eigenvector components of the PC1 obtained from conPCA of the YTH‐A3 RNA. (D) Representative structures of the YTH‐A3 RNA discriminated by the PC1. In (B) and (D), those important contacts are highlighted and the corresponding residues and m^6^A3/A3 are drawn in ball‐and‐stick.

ConPCA [29] was performed on the aMD trajectories of the YTH‐m^6^A3 RNA, using twenty contacts between m^6^A3 and the YTH domain. Fig.5 shows the eigenvector components of the PC1 in a descending order of the absolute values, which indicate the weights of these contacts to the conformational changes of the complex. Since the recognition loop remains closed in the YTH‐m^6^A3 RNA, only the contact between M437 and m^6^A3 has a large weight ( Fig.5). Representative structures discriminated by the PC1 ( Fig.5) show that M437 is in a transition between a “in” and an “out” conformation. R478 also has a large weight, but it is not in the recognition loop. From conPCA results of the YTH‐A3 RNA, the first six contacts with the largest weights are all formed between the hydrophobic residues (W431, P434, L433, V432, M437, and L442) and the A3 ( Fig.5). Their eigenvector components of PC1 are all negative which mean they form or break simultaneously during the simulation. When looking at the representative structures along the PC1 ( Fig.5), the hydrophobic residues interact with the A3 initially. During the aMD simulation, the contacts are all broken, so the recognition loop uncovers the binding pocket and the A3 is exposed. The results again support that hydrophobic interactions play a critical role in the binding between the YTH domain and the RNA. These hydrophobic residues in the recognition loop bind less favorably to the A3 than to the m^6^A3.

## DISCUSSION

To the best of our knowledge, detailed structural and dynamic information of the YTH‐RNA complex is still lacking. In this work, we present an atomic description of the YTH‐m^6^A3/A3 complexes using aMD simulations. The results are in reasonable agreement with the experimental data [18]. The YTH‐m^6^A3 RNA is more stable than the YTH‐A3 RNA. The recognition loop remains closed and covers the binding pocket in the methylated complex. However, in the non‐methylated complex, the loop has a large movement that makes the binding pocket exposed, and thus the A3 can be released.

The binding mechanism between the protein and the RNA can be described by either “conformational selection” [30] or “induced fit” [31]. A solution structure of the apo YTH from *Homo sapiens*, which has a sequence identity of 86% with the YTH studied in this work, adopts a closed conformation (PDB code: 2YUD). This seems to support a “induced fit” mechanism for RNA binding. To address this issue, aMD simulations of the apo YTH were performed. The apo YTH can sample a large conformational space with diverse conformations, and there is an intrinsic close‐open transition of the recognition loop in the aMD trajectories. Upon ligand binding (m^6^A3 RNA or A3 RNA), the YTH domain can only sample a limited region. The conformations of the YTH‐m^6^A3 RNA are relatively stable and keep closed, whereas the YTH‐A3 RNA can get access to the open conformation. Our findings suggest a “conformational selection” mechanism between YTH and RNA.

By free energy decomposition using the MM/GBSA method, it has been found that two regions of hydrophobic residues in the YTH domain contribute to the favorable binding of the m^6^A3 RNA. We have mutated two residues (L383A and M437A), and the aMD simulations indicate that the mutYTH‐m^6^A3 RNA becomes a little open with less favorable binding than the YTH‐m^6^A3 RNA. Additional virtual mutations can be done in the future. For example, conPCA on the YTH‐A3 RNA indicates that contacts between W431 and A3 has the largest contribution. Li *et al*. also mentioned an “aromatic cage” formed by Trp [24].

Our findings may be helpful to interpret the binding mechanism of the YTH domain as a m^6^A reader. The observation of “conformational selection” of the YTH‐RNA binding has raised some more questions. We have sampled a close‐open transition of the recognition loop during the aMD simulations. By following the standard reweighting procedure of aMD, the free energy difference between the closed and the open state shown on the PCA essential subspace (Supplementary Fig. S1) is about ‒6.0 kcal mol^‒1^ in the apo YTH and the energy barrier of the transition state is about 12.3 kcal mol^‒1^. In the YTH‐A3 RNA, the free energy difference between the two states is about ‒12.1 kcal mol^‒1^, and the energy barrier is about 14.8 kcal mol^‒1^. However, it should be noted that there is still a sampling issue although we have conducted three independent 1‐µs aMD simulations. Future work would be calculating the free energy difference more accurately using other advanced techniques [32].

## MATERIALS AND METHODS

### Simulated systems

The *Rattus norvegicus* protein YT521‐B ( YTHDC1) contains a YTH domain (residues 347‒502) [16]. A complex structure of the YTH domain with a N^6^‐methylated RNA (5′‐UGm^6^ACAC‐3′) was solved by solution nuclear magnetic resonance (NMR) [18]. In this work, we used the first model of this NMR structure (PDB entry 2MTV) as the starting structure of simulations, and the system is denoted as YTH‐m^6^A3 RNA. We then changed m^6^A3 to a non‐methylated adenine, and built a system called YTH‐A3 RNA. By removing RNA from YTH‐m^6^A3 RNA, we built the apo YTH. After mutating L383 and M437 to ALA in the YTH‐m^6^A3 RNA, the mutYTH‐m^6^A3 RNA was obtained.

### Conventional molecular dynamics (cMD) simulations

cMD simulations were carried out by the Amber14 package [33]. Each system was built in the tleap module [34] using the ff14SB force field [35] for protein and bsc0χOL3 force field for RNA [36,37]. Parameters for the m^6^A3 were from [38]. The structure was immersed into a truncated octahedral box that extended 10 Å away from the solute border, using the TIP3P water model [39] and periodic boundary conditions. One Cl^‒^ was added in the box to neutralize the system. Therefore, the total number of atoms was 22,552 in the YTH‐m^6^A3 RNA, 22,555 in the YTH‐A3 RNA, 19,702 in the apo YTH, and 22,455 in the mutYTH‐m^6^A3 RNA. The waters and ions were initially minimized for 2000 steps using the steepest descent method for the first 1000 steps and then the conjugate gradient algorithm for the last 1000 steps, with the position of protein and RNA fixed (force constant was 500 kcal mol^‒1^ Å^‒2^). In the second energy minimization stage, the restraints on the protein and RNA were removed. This stage was conducted for 2500 steps, using the steepest descent method in the first 1000 steps and then the conjugate gradient algorithm for the last 1500 steps. After that, a heat‐up MD was run at a constant volume. The system was heated from 0 to 300 K for 100 ps with a weak restraint of 10 kcal mol^‒1^ Å^−2^ on the solute. Then, free MD simulations were carried out under the NPT condition. Temperature was regulated using the Langevin dynamics [40,41] with a collision frequency of 1.0 ps^−1^. Pressure was controlled with isotropic position scaling at 1 bar with a relaxation time of 2.0 ps. All of the bonds involving hydrogen atoms were constrained using the SHAKE algorithm [42]. A 2 fs integration step was used. The long‐range electrostatic interactions were calculated using PME method [43] with a 10 Å cutoff for the range‐limited non‐bonded interactions. Three independent 100‐ns cMD simulations were performed for each system.

### Accelerated molecular dynamics (aMD) simulations

aMD simulations enhance conformational sampling of a biomolecule by adding a boost potential Δ $V_{\left(r\right)}$ to the original potential $V_{\left(r\right)}$ when the latter is below a threshold energy E [44].

(1) $$\left\{ \begin{array}{ll} V_{\left(r\right)}^{*}=V_{\left(r\right)} & V_{\left(r\right)}\gg E,\\ V_{\left(r\right)}^{*}=V_{\left(r\right)}+\Delta V_{\left(r\right)} & V_{\left(r\right)}<E. \end{array}\right.$$

In the simplest form, the boost potential is given by

(2) $$\Delta V_{\left(r\right)}=\frac{{(E-(V_{\left(r\right)})\;)}^{2}}{\alpha +E-V_{\left(r\right)}},$$

which can flat the energy potential surface and induce the conformational transition between the low‐energy states when the acceleration factor α decreases.

Boosting potentials are often applied to both the total potential and the dihedral energy terms. Here, we used 100 ns cMD trajectories to estimate the aMD input parameters. For YTH‐m^6^A3 RNA with 156 residues and 6 nucleotides, the average total potential energy is ‒68,357 kcal mol^−1^ and the average dihedral energy is 2016 kcal mol^‒1^. The following parameters were set based on the above information:

E_tot_= ‒68,357 kcal mol^‒1^ + (0.16 kcal mol^‒1^ atom^‒1^ × 22,552 atoms) ≈ ‒64,749 kcal mol^‒1^

α_tot_ = (0.2 kcal mol^‒1^ atom^‒1^ × 22,552 atoms) ≈ 4510 kcal mol^‒1^

E_dih_ = 2016 kcal mol^‒1^ + (3.5 kcal mol^‒1^ residue^‒1^ × 162 residues) ≈ 2583 kcal mol^‒1^

α_dih_ = 0.2 × (3.5 kcal mol^‒1^ residues^‒1^ × 162 residues) ≈ 113 kcal mol^‒1^

For the YTH‐A3 RNA, the average total potential energy is ‒66,609 kcal mol^‒1^ and the average dihedral energy was 1949 kcal mol^‒1^. The aMD parameters were set as follow.

E_tot_ = ‒66,609 kcal mol^‒1^ + (0.16 kcal mol^‒1^ atom^‒1^ × 22,555 atoms) ≈ ‒63,001 kcal mol^‒1^

α_tot_ = (0.2 kcal mol^‒1^ atom^‒1^ × 22,555 atoms) ≈ 4511 kcal mol^‒1^

E_dih_ = 1949 kcal mol^‒1^ + (3.5 kcal mol^‒1^ residue^‒1^ × 162 residues) ≈ 2516 kcal mol^‒1^

α_dih_ = 0.2 × (3.5 kcal mol^‒1^ residues^‒1^ × 162 residues) ≈ 113 kcal mol^‒1^

For the apo YTH, the average total potential energy is ‒59,185 kcal mol^‒1^ and the average dihedral energy was 1933 kcal mol^‒1^. The aMD parameters were set as follow.

E_tot_ = ‒59,185 kcal mol^‒1^ + (0.16 kcal mol^‒1^ atom^‒1^ × 19702 atoms) ≈‒56,033 kcal mol^‒1^

α_tot_ = (0.2 kcal mol^‒1^ atom^‒1^ × 19,702 atoms) ≈ 3940 kcal mol^‒1^

E_dih_ = 1933 kcal mol^‒1^ + (3.5 kcal mol^‒1^ residue^‒1^ × 156 residues) ≈ 2479 kcal mol^‒1^

α_dih_ = 0.2 × (3.5 kcal mol^‒1^ residues^‒1^ × 156 residues) ≈ 109 kcal mol^‒1^

For the mutYTH‐m^6^A3 RNA, the average total potential energy is ‒68,088 kcal mol^‒1^ and the average dihedral energy was 2042 kcal mol^‒1^. The aMD parameters were set as follow.

E_tot_ = ‒68,087 kcal mol^‒1^ + (0.16 kcal mol^‒1^ atom^‒1^ × 22,455 atoms) ≈ ‒64,494 kcal mol^‒1^

α_tot_= (0.2 kcal mol^‒1^ atom^‒1^ × 22,455 atoms) ≈ 4491 kcal mol^‒1^

E_dih_ = 2042 kcal mol^‒1^ + (3.5 kcal mol^‒1^ residue^‒1^ × 162 residues) ≈ 2609 kcal mol^‒1^

α_dih_ = 0.2 × (3.5 kcal mol^‒1^ residues^‒1^ × 162 residues) ≈ 113 kcal mol^‒1^

All the other parameters were the same as those in the cMD simulations. The aMD simulations were performed starting from the final structure of the heat‐up procedure, that is to say, the initial conformations of aMD and cMD are the same. To obtain statistically more meaningful results, three independent 1‐µs aMD simulation were run for each system.

### Molecular mechanics generalized Born surface area (MM/GBSA)

MM/GBSA [28] was used to estimate binding free energies between the protein and the RNA, and per‐residue free energy decomposition [45,46], from the aMD trajectories. The script MMPBSA.py.MPI was used.

### Principal component analysis (PCA)

In PCA, the correlated internal motion with *N* degrees of freedom can be described by a covariance matrix [26],

(6) $$\sigma_{mn}=\left\langle \left(r_{m}-\left\langle r_{m}\right\rangle \right)\left(r_{n}-\left\langle r_{n}\right\rangle \right)\right\rangle ,$$

where $r_{1}$ ,_···_, $r_{N}$ mean the Cartesian coordinates and ⟨⋅⋅⋅⟩ represents the ensemble average. Diagonalization of this covariance matrix can obtain 3 *N*−6 eigenvectors (called PCA modes) with non‐zero eigenvalues that represent fluctuations of corresponding modes. The PCA modes with the largest eigenvalues (denoted as essential modes) usually describe functionally relevant collective motions of the system.

For each system, the combined aMD trajectories contain 3,000 conformations. The C_α_ atoms in the protein were used to construct the covariance matrix. Projection of a trajectory on the essential PCA modes can be used to visualize conformational sampling during the simulation.

### Contact‐based principal component analysis (ConPCA)

Interactions in the native structure are likely to play an important role in structural dynamics. First, we determined the native contacts between the m^6^A3 and the YTH domain from the NMR structure. A contact is defined when the heavy‐atom distance between a residue *i* and a nucleotide *j* is less than 6.0 Å. Then, we calculated the contact distance D_*ij*_ for each conformation in a trajectory using the g_mindist program in the Gromacs‐4.5.5 package [47]. The data of distances were used as input of ConPCA [29]. Employing the definition of Eq. (6), we construct the following covariance matrix.

(7) $$\sigma_{ij}=\left\langle \left(D_{i}-\left\langle D_{i}\right\rangle \right)\left(D_{j}-\left\langle D_{j}\right\rangle \right)\right\rangle .$$

## SUPPLEMENTARY MATERIALS

The supplementary materials can be found online with this article at https://doi.org/10.15302/J‐QB‐022‐0297.

## COMPLIANCE WITH ETHICS GUIDELINES

The authors Mingwei Li, Guanglin Chen and Zhiyong Zhang declare that they have no conflict of interests.

This article does not contain any studies with human or animal subjects performed by any of the authors.

## Supporting information

Supplementary Information

## References

[1] Meyer, K. D. Jaffrey, S. (2014). The dynamic epitranscriptome: N^6^‐methyladenosine and gene expression control. Nat. Rev. Mol. Cell Biol., 15: 313–326 10.1038/nrm3785 24713629 PMC4393108

[2] Fu, Y. , Dominissini, D. , Rechavi, G. (2014). Gene expression regulation mediated through reversible m^6^A RNA methylation. Nat. Rev. Genet., 15: 293–306 10.1038/nrg3724 24662220

[3] Meyer, K. D. , Saletore, Y. , Zumbo, P. , Elemento, O. , Mason, C. E. Jaffrey, S. (2012). Comprehensive analysis of mRNA methylation reveals enrichment in 3′ UTRs and near stop codons. Cell, 149: 1635–1646 10.1016/j.cell.2012.05.003 22608085 PMC3383396

[4] Dominissini, D. , Moshitch‐Moshkovitz, S. , Schwartz, S. , Salmon‐Divon, M. , Ungar, L. , Osenberg, S. , Cesarkas, K. , Jacob‐Hirsch, J. , Amariglio, N. , Kupiec, M. et al.. (2012). Topology of the human and mouse m^6^A RNA methylomes revealed by m^6^A‐seq. Nature, 485: 201–206 10.1038/nature11112 22575960

[5] Liu, N. , Zhou, K. I. , Parisien, M. , Dai, Q. , Diatchenko, L. (2017). N^6^‐methyladenosine alters RNA structure to regulate binding of a low‐complexity protein. Nucleic Acids Res., 45: 6051–6063 10.1093/nar/gkx141 28334903 PMC5449601

[6] Wang, X. , Lu, Z. , Gomez, A. , Hon, G. C. , Yue, Y. , Han, D. , Fu, Y. , Parisien, M. , Dai, Q. , Jia, G. et al.. (2014). N^6^‐methyladenosine‐dependent regulation of messenger RNA stability. Nature, 505: 117–120 10.1038/nature12730 24284625 PMC3877715

[7] Chandola, U. , Das, R. (2015). Role of the N^6^‐methyladenosine RNA mark in gene regulation and its implications on development and disease. Brief. Funct. Genomics, 14: 169–179 10.1093/bfgp/elu039 25305461

[8] Liu, J. , Yue, Y. , Han, D. , Wang, X. , Fu, Y. , Zhang, L. , Jia, G. , Yu, M. , Lu, Z. , Deng, X. et al.. (2014). A METTL3‐METTL14 complex mediates mammalian nuclear RNA N^6^‐adenosine methylation. Nat. Chem. Biol., 10: 93–95 10.1038/nchembio.1432 24316715 PMC3911877

[9] Lee, M. , Kim, B. Kim, V. (2014). Emerging roles of RNA modification: m^6^A and U‐tail. Cell, 158: 980–987 10.1016/j.cell.2014.08.005 25171402

[10] Jia, G. , Fu, Y. , Zhao, X. , Dai, Q. , Zheng, G. , Yang, Y. , Yi, C. , Lindahl, T. , Pan, T. , Yang, Y. G. et al.. (2011). N^6^‐methyladenosine in nuclear RNA is a major substrate of the obesity‐associated FTO. Nat. Chem. Biol., 7: 885–887 10.1038/nchembio.687 22002720 PMC3218240

[11] Aik, W. , Scotti, J. S. , Choi, H. , Gong, L. , Demetriades, M. , Schofield, C. J. McDonough, M. (2014). Structure of human RNA N^6^‐methyladenine demethylase ALKBH5 provides insights into its mechanisms of nucleic acid recognition and demethylation. Nucleic Acids Res., 42: 4741–4754 10.1093/nar/gku085 24489119 PMC3985658

[12] Wang, X. , Zhao, B. S. , Roundtree, I. A. , Lu, Z. , Han, D. , Ma, H. , Weng, X. , Chen, K. , Shi, H. (2015). N^6^‐methyladenosine modulates messenger RNA translation efficiency. Cell, 161: 1388–1399 10.1016/j.cell.2015.05.014 26046440 PMC4825696

[13] Zhou, J. , Wan, J. , Gao, X. , Zhang, X. , Jaffrey, S. R. Qian, S. (2015). Dynamic m^6^A mRNA methylation directs translational control of heat shock response. Nature, 526: 591–594 10.1038/nature15377 26458103 PMC4851248

[14] Zhang, Z. , Theler, D. , Kaminska, K. H. , Hiller, M. , de la Grange, P. , Pudimat, R. , Rafalska, I. , Heinrich, B. , Bujnicki, J. M. , Allain, F. H. T. et al.. (2010). The YTH domain is a novel RNA binding domain. J. Biol. Chem., 285: 14701–14710 10.1074/jbc.M110.104711 20167602 PMC2863249

[15] Hsu, P. J. , Zhu, Y. , Ma, H. , Guo, Y. , Shi, X. , Liu, Y. , Qi, M. , Lu, Z. , Shi, H. , Wang, J. et al.. (2017). YTHDC2 is an N^6^‐methyladenosine binding protein that regulates mammalian spermatogenesis. Cell Res., 27: 1115–1127 10.1038/cr.2017.99 28809393 PMC5587856

[16] Imai, Y. , Matsuo, N. , Ogawa, S. , Tohyama, M. (1998). Cloning of a gene, YT521, for a novel RNA splicing‐related protein induced by hypoxia/reoxygenation. Brain Res. Mol. Brain Res., 53: 33–40 10.1016/S0169-328X(97)00262-3 9473574

[17] Hartmann, A. M. , Nayler, O. , Schwaiger, F. W. , Obermeier, A. (1999). The interaction and colocalization of Sam68 with the splicing‐associated factor YT521‐B in nuclear dots is regulated by the Src family kinase p59^fyn^. Mol. Biol. Cell, 10: 3909–3926 10.1091/mbc.10.11.3909 10564280 PMC25688

[18] Theler, D. , Dominguez, C. , Blatter, M. , Boudet, J. Allain, F. H. (2014). Solution structure of the YTH domain in complex with N^6^‐methyladenosine RNA: a reader of methylated RNA. Nucleic Acids Res., 42: 13911–13919 10.1093/nar/gku1116 25389274 PMC4267619

[19] Luo, S. (2014). Molecular basis for the recognition of methylated adenines in RNA by the eukaryotic YTH domain. Proc. Natl. Acad. Sci. USA, 111: 13834–13839 10.1073/pnas.1412742111 25201973 PMC4183320

[20] Xu, C. , Wang, X. , Liu, K. , Roundtree, I. A. , Tempel, W. , Li, Y. , Lu, Z. , He, C. (2014). Structural basis for selective binding of m^6^A RNA by the YTHDC1 YTH domain. Nat. Chem. Biol., 10: 927–929 10.1038/nchembio.1654 25242552

[21] Govindaraju, G. , Kadumuri, R. V. , Sethumadhavan, D. V. , Jabeena, C. A. , Chavali, S. (2020). N^6^‐adenosine methylation on mRNA is recognized by YTH2 domain protein of human malaria parasite Plasmodium falciparum. Epigenet. Chromatin, 13: 33 10.1186/s13072-020-00355-7 PMC7457798 32867812

[22] Li, F. , Zhao, D. , Wu, J. (2014). Structure of the YTH domain of human YTHDF2 in complex with an m^6^A mononucleotide reveals an aromatic cage for m^6^A recognition. Cell Res., 24: 1490–1492 10.1038/cr.2014.153 25412658 PMC4260351

[23] Xu, C. , Liu, K. , Ahmed, H. , Loppnau, P. , Schapira, M. (2015). Structural basis for the discriminative recognition of N^6^‐methyladenosine RNA by the human YT521‐B homology domain family of proteins. J. Biol. Chem., 290: 24902–24913 10.1074/jbc.M115.680389 26318451 PMC4598999

[24] Li, Y. , Bedi, R. K. , Wiedmer, L. , Sun, X. , Huang, D. (2021). Atomistic and thermodynamic analysis of N^6^‐methyladenosine (m^6^A) recognition by the reader domain of YTHDC1. J. Chem. Theory Comput., 17: 1240–1249 10.1021/acs.jctc.0c01136 33472367

[25] Li, Y. , Bedi, R. K. , Moroz‐Omori, E. V. (2020). Structural and dynamic insights into redundant function of YTHDF proteins. J. Chem. Inf. Model., 60: 5932–5935 10.1021/acs.jcim.0c01029 33073985

[26] Amadei, A. , Linssen, A. B. M. Berendsen, H. J. (1993). Essential dynamics of proteins. Proteins, 17: 412–425 10.1002/prot.340170408 8108382

[27] Berendsen, H. J. C. (2000). Collective protein dynamics in relation to function. Curr. Opin. Struct. Biol., 10: 165–169 10.1016/S0959-440X(00)00061-0 10753809

[28] Miller, B. R. McGee, T. D. Swails, J. M. , Homeyer, N. , Gohlke, H. Roitberg, A. (2012). MMPBSA. py: An efficient program for end‐state free energy calculations. J. Chem. Theory Comput., 8: 3314–3321 10.1021/ct300418h 26605738

[29] Ernst, M. , Sittel, F. (2015). Contact‐ and distance‐based principal component analysis of protein dynamics. J. Chem. Phys., 143: 244114 10.1063/1.4938249 26723658

[30] Koshland, D. E. (1958). Application of a theory of enzyme specificity to protein synthesis. Proc. Natl. Acad. Sci. USA, 44: 98–104 10.1073/pnas.44.2.98 16590179 PMC335371

[31] Williamson, J. (2000). Induced fit in RNA‐protein recognition. Nat. Struct. Biol., 7: 834–837 10.1038/79575 11017187

[32] Liao, Q. (2020). Enhanced sampling and free energy calculations for protein simulations. In: Progress in Molecular Biology and Translational Science, 177 32145945 10.1016/bs.pmbts.2020.01.006

[33] Pearlman, D. A. , Case, D. A. , Caldwell, J. W. , Ross, W. S. , Cheatham, T. E. Debolt, S. , Ferguson, D. , Seibel, G. (1995). Amber, a package of computer‐programs for applying molecular mechanics, normal‐mode analysis, molecular‐dynamics and free‐energy calculations to simulate the structural and energetic properties of molecules. Computer Physics Communications. Comput. Phys. Commun., 91: 1–41 10.1016/0010-4655(95)00041-D

[34] Case, D. A. , Cheatham, T. E. Darden, T. , Gohlke, H. , Luo, R. , Merz, K. M. Onufriev, A. , Simmerling, C. , Wang, B. Woods, R. (2005). The Amber biomolecular simulation programs. J. Comput. Chem., 26: 1668–1688 10.1002/jcc.20290 16200636 PMC1989667

[35] Maier, J. A. , Martinez, C. , Kasavajhala, K. , Wickstrom, L. , Hauser, K. E. (2015). ff14SB: Improving the accuracy of protein side chain and backbone parameters from ff99SB. J. Chem. Theory Comput., 11: 3696–3713 10.1021/acs.jctc.5b00255 26574453 PMC4821407

[36] Cornell, W. D. , Cieplak, P. , Bayly, C. I. , Gould, I. R. , Merz, K. M. , Ferguson, D. M. , Spellmeyer, D. C. , Fox, T. , Caldwell, J. W. Kollman, P. (1995). A 2nd generation force‐field for the simulation of proteins, nucleic‐acids, and organic‐molecules. J. Am. Chem. Soc., 117: 5179–5197 10.1021/ja00124a002

[37] Otyepka, M. , Sponer, J. , Mládek, A. , Cheatham, T. E. (2011). Refinement of the Cornell *et al*. nucleic acids force field based on reference quantum chemical calculations of glycosidic torsion profiles. J. Chem. Theory Comput., 7: 2886–2902 10.1021/ct200162x 21921995 PMC3171997

[38] Aduri, R. , Psciuk, B. T. , Saro, P. , Taniga, H. , Schlegel, H. B. (2007). AMBER force field parameters for the naturally occurring modified nucleosides in RNA. J. Chem. Theory Comput., 3: 1464–1475 10.1021/ct600329w 26633217

[39] Mark, P. (2001). Structure and dynamics of the TIP3P, SPC, and SPC/E water models at 298 K. J. Phys. Chem. A, 105: 9954–9960 10.1021/jp003020w

[40] Pastor, R. W. , Brooks, B. R. (2006). An analysis of the accuracy of Langevin and molecular dynamics algorithms. Mol. Phys., 65: 1409–1419 10.1080/00268978800101881

[41] Feller, S. E. , Zhang, Y. H. , Pastor, R. W. Brooks, B. (1995). Constant‐pressure molecular‐dynamics simulation―the Langevin piston method. J. Chem. Phys., 103: 4613–4621 10.1063/1.470648

[42] Forester, T. R. (2000). SHAKE, rattle, and roll: Efficient constraint algorithms for linked rigid bodies. J. Comput. Chem., 21: 157–157 10.1002/(SICI)1096-987X(20000130)21:2<157::AID-JCC7>3.0.CO;2-2

[43] Darden, T. , York, D. (1993). Particle mesh Eewald: an *N*. Log(N) method for Ewald sums in large systems. J. Chem. Phys., 98: 10089–10092 10.1063/1.464397

[44] Hamelberg, D. , Mongan, J. McCammon, J. (2004). Accelerated molecular dynamics: a promising and efficient simulation method for biomolecules. J. Chem. Phys., 120: 11919–11929 10.1063/1.1755656 15268227

[45] Kollman, P. A. , Massova, I. , Reyes, C. , Kuhn, B. , Huo, S. , Chong, L. , Lee, M. , Lee, T. , Duan, Y. , Wang, W. et al.. (2000). Calculating structures and free energies of complex molecules: combining molecular mechanics and continuum models. Acc. Chem. Res., 33: 889–897 10.1021/ar000033j 11123888

[46] Massova, I. Kollman, P. (2000). Combined molecular mechanical and continuum solvent approach (MM‐PBSA/GBSA) to predict ligand binding. Perspect. Drug Discov. Des., 18: 113–135 10.1023/A:1008763014207

[47] Pronk, S. , Páll, S. , Schulz, R. , Larsson, P. , Bjelkmar, P. , Apostolov, R. , Shirts, M. R. , Smith, J. C. , Kasson, P. M. , van der Spoel, D. et al.. (2013). GROMACS 4. 5: a high‐throughput and highly parallel open source molecular simulation toolkit. Bioinformatics, 29: 845–854 10.1093/bioinformatics/btt055 23407358 PMC3605599

