# Supplementary material for: Structural and dynamic properties of the YTH domain in complex with N6‐methyladenosine RNA studied by accelerated molecular dynamics simulations
Source: Quant Biol. 2023 Mar 1;11(1):72–81. doi: 10.15302/J-QB-022-0297 (PMC12807427; doi:10.15302/J-QB-022-0297)
Supplement: Supplementary file 1 — Supplementary Information [file QUB2-11-72-s001.pdf]

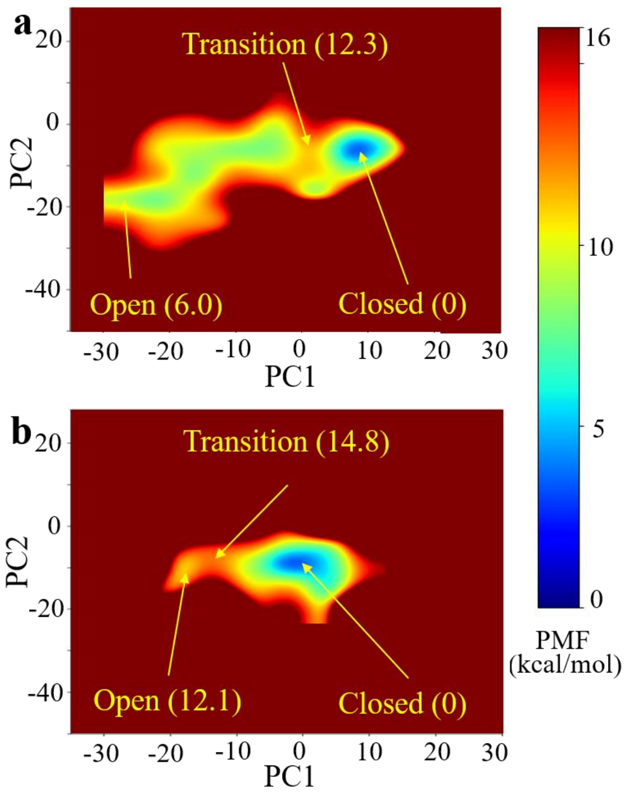

Figure S1: Two-dimensional free energy profiles on the principal components (PC1 and PC2) in (a) one aMD simulation of the apo YTH, and (b) one aMD simulation of the YTH-A3 RNA.
